# Central and Peripheral NPY Age-Related Regulation: A Comparative Analysis in Fish Translational Models

**DOI:** 10.3390/ijms23073839

**Published:** 2022-03-30

**Authors:** Daniela Giaquinto, Elena De Felice, Chiara Attanasio, Antonio Palladino, Valentina Schiano, Ernesto Mollo, Carla Lucini, Paolo de Girolamo, Livia D’Angelo

**Affiliations:** 1 Department Veterinary Medicine and Animal Production, University of Naples Federico II, Via F. Delpino 1, 80137 Naples, Italy; daniela.giaquinto@unina.it (D.G.); chiara.attanasio@unina.it (C.A.); valentinaschiano8742@gmail.com (V.S.); carla.lucini@unina.it (C.L.); degirola@unina.it (P.d.G.); 2 School of Biosciences and Veterinary Medicine, University of Camerino, Via Gentile III da Varano, 62032 Camerino, Italy; elena.defelice@unicam.it; 3 Department Agricultural Sciences, University of Naples Federico II, Via Università 100, 80055 Portici, Italy; antonio.palladino@unina.it; 4 Consiglio Nazionale delle Ricerche-Istituto di Chimica Biomolecolare (CNR-ICB), Via Campi Flegrei 34, 80078 Pozzuoli, Italy; emollo@icb.cnr.it

**Keywords:** neuropeptides, brain, aging, African turquoise killifish, zebrafish, brain, gut

## Abstract

NPY is among the most abundant neuropeptides in vertebrate brain and is primarily involved in the regulation of food intake. The NPY system is also associated with the aging process showing beneficial effects on neuronal survival via autophagy modulation. Here, we explore the age-related regulation of NPY in the brain and foregut of the shortest- and longest-lived fish species, Nothobranchius furzeri and Danio rerio, respectively. These two research models, despite some similarities, display profound biological differences making them attractive vertebrates to elucidate the mechanisms underlying the regulation of neuropeptide synthesis and function. It is noteworthy that in both fish species only Npya has been identified, while in the other teleosts two classes of NPY (Npya and Npyb) have been annotated. Our findings document that in both species: (i) NPY is centrally regulated; (ii) NPY levels increase in the brain during aging; (iii) NPY is localized in the enteroendocrine cells as well as in the myenteric plexus and drastically decreases in old animals. According to our data, the age-related regulation in the gut resembles that described in other vertebrate species while the increased levels in the brain offer the unique possibility to explore the role of NPY in model organisms to develop future experimental and translatable approaches.

## 1. Introduction

A recent article published in *The Lancet* [1] investigated life expectancy in 35 countries around the world reporting a projected increase ranging from 65% for women to 85% for men by 2030.

From the biological perspective, aging is the result of the accumulation of a wide variety of damage at both the molecular and cellular level that, occurring over time, induces a gradual physical and mental decline along with an increasing risk of disease.

In this scenario, the role of research oriented to unravel the biological mechanisms underlying aging is even more crucial than before.

In an elegant article Wyss-Coray [2] illustrated an interesting point of view regarding brain aging based on the high incidence of neurodegenerative diseases and the infrequency of disease-free brains in the aged population. This author supported the hypothesis that physiological aging forms a kind of continuum with neurodegenerative disorders which is accelerated by stochastic factors that interact variously in inducing the onset of specific diseases.

Growing evidence suggests that the NPY system is associated with the aging process with beneficial effects on neuronal survival exerted by modulating autophagy. Caloric restriction, NPY, and ghrelin, indeed, stimulate autophagy through PI3K/AKT/MTOR inhibition and ERK1/2-MAPK activation [3].

NPY is a 36 amino acid peptide originally isolated from the porcine brain in 1982 [4]. In mammals, NPY is one of the most abundant neuropeptides in the peripheral (PNS) and central (CNS) nervous systems, but also in other peripheral tissues [5]. In the CNS, NPY fulfils several physiological functions related to feeding behavior, learning and memory, anxiety control, and circadian rhythm, to name just a few. Therefore, when dysfunctional, it may be involved in the onset of the related pathologies [6]. The association of the NPY system with aging and even its role in lifespan determination has been widely documented in the literature. In particular, NPY has been reported to impact on six hallmarks of aging, namely loss of proteostasis, stem cell exhaustion, altered intercellular communication, deregulated nutrient sensing, cellular senescence, and mitochondrial dysfunction [7]. In this context, it has been shown that transgenic rats overexpressing NPY live longer [8]; this effect is associated with lower blood pressure at the baseline and during stress along with decreased levels of catecholamines and lower sympathetic drive to the periphery.

Moreover, NPY Y2-receptor knockout (KO) mice displayed learning deficits and memory deterioration during a Morris water maze and object recognition task. These results clearly indicate that Y2 receptors might play a significant role in spatial reference memory and nonspatial working memory processing [9]. In contrast, in aged rodents as well as in brain samples from individuals with neurodegenerative disease, the expression levels of NPY and NPY receptors had decreased in several brain areas [7]. Further, it has been shown both in humans and rodent models that NPY levels are altered in some neurodegenerative disorders, suggesting an involvement of NPY in their onset and a neuroprotective role of NP exerted by the stimulation of neural stem cell proliferation and trophic support, besides the enhancement of autophagy and the inhibition of excitotoxicity and neuroinflammation [10]. It is worth noting that even old-onset caloric restriction maintains the total number of NPY neurons in the hippocampus, the brain area that during aging is physiologically associated with a loss of these neurons [11]. It is in this context that we have focused our interest on exploring the age-related changes of NPY in the African turquoise killifish (*Nothobranchius furzeri*), an emerging but now also well-recognized, model organism for the study of aging processes. In this teleost fish, displaying the shortest lifespan ever reported in captivity [12,13,14], a rapid growth is associated with a relatively early expression of aging phenotypes at multiple levels (morphological, physiological, and behavioral) [15,16,17]. In this work we aim to analyze and compare the age-related central and peripheral regulation of NPY in the shortest- and longest-lived fish species, turquoise killifish and zebrafish (*Danio rerio*), respectively. These two species despite some anatomical correspondences such as a comparable embryo size and digestive system organization as well as similarities in breeding methodologies display significant biological differences: (i) embryonic development, which is slower (∼2–3 weeks) in *N. furzeri* compared to zebrafish (∼2–3 days) [18,19]; (ii) growth and sexual maturation time in controlled laboratory conditions is faster in the turquoise killifish (5.5–8 weeks) than in zebrafish (12–13 weeks) [20,21]; (iii) lifespan, lasting 4–6 months in the turquoise killifish and up to 5 years in zebrafish.

In teleost fish, NPY is primarily implicated in the control of food intake [22], interacting with other orexigenic peptides, i.e., orexin A and B and galanin, in an interdependent and coordinated manner [22]. On the other hand, the action of NPY and orexins is modulated by the synergistic effects of cholecystokinin and cocaine- and amphetamine-regulated transcripts, two potent anorexigenic factors in fish [23]. The hypothalamus is the central region responsible for the synthesis and release of these peptides upon the integration of information of a different nature such as levels of nutrients and hormones as well as circadian signals [23]. It is noteworthy that in fish NPY is synthesized by hypothalamic neurons also releasing Agouti Related Protein (AgRP), an antagonist for the anorexigenic melanocortin receptors [22]. A plethora of studies have demonstrated the evolutionary conserved orexigenic function of NPY in fish [23,24]. Interestingly, in addition to the pivotal role in regulating food intake, it has been recently demonstrated that NPY is involved in numerous and different functions of fish, such as psychomotor activity [25], circadian rhythmicity [26], anxiety-like behaviors [27], and the recovery of motor function following spinal cord injury [28]. However, the role of NPY in fish species during aging is still unexplored. In addition, both in turquoise killifish and zebrafish only Npya has been identified [29,30], unlike other Teleosts in which two classes of NPY (Npya and Npyb) have been reported. In this setting, we aim to unravel the age-related mechanisms involving the NPY-ergic system in vertebrates by comparing the variation in the expression levels of NPY mRNA in both the brain and the foregut of the two species as well as by describing the pattern of NPY distribution over aging.

## 2. Results

### 2.1. NPY mRNA Expression Levels in the Brain and Gut during Aging

**Figure 1:**
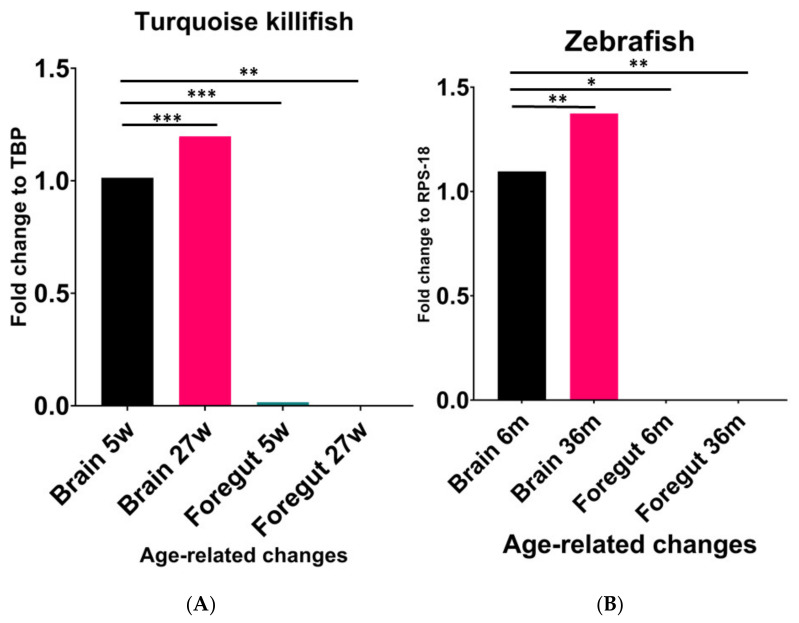
Comparative expression levels of NPY mRNA in the brain and foregut of young and old turquoise killifish (**A**) and zebrafish (**B**). (**A**) Significant increase in NPY mRNA in the brain of 27 wph animals compared to 5 wph ones. Higher expression levels in the brain of animals at 5 wph compared to the levels detected in the foregut of the same animals. (**B**) Significant increase in NPY mRNA in the brain of 36-month-old zebrafish compared to 6-month-old animals. Significant decrease in the foregut compared to the brain either in young or old specimens. * (*p* < 0.5), ** (*p* < 0.05), *** (*p* < 0.001).

We first compared the expression levels of NPY mRNA in the brain and foregut of turquoise killifish at 5 wph and 27 wph. Similar to previous results [30], we observed that the NPY expression levels significantly increased from young to old animals (Figure 1A). A significant increase in NPY was observed in the brains of old animals when compared to the brains of young animals (*p* < 0.0002). In contrast, higher expression levels were detected in the brain of animals at 5 wph compared to the levels in the gut of the same animals (*p* < 0.0002), as well as in the gut of animals at 27 wph (*p* < 0.0063) (Figure 1). A similar age-associated change in the NPY expression levels was detected in zebrafish (Figure 1B). Specifically, we observed a significant increase in NPY mRNA in the brain of 36-month-old zebrafish when compared to 6-month-old animals (*p* < 0.0024), and a significant decrease in the gut of young animals compared to the brain of the same animal (*p* < 0.170) as well as when comparing the levels of the neuropeptide in the gut of old animals (*p* < 0.0025) (Figure 1B).

### 2.2. Immunoreactivity to NPY in the Brain of Turquoise Killifish and Zebrafish

**Figure 2:**
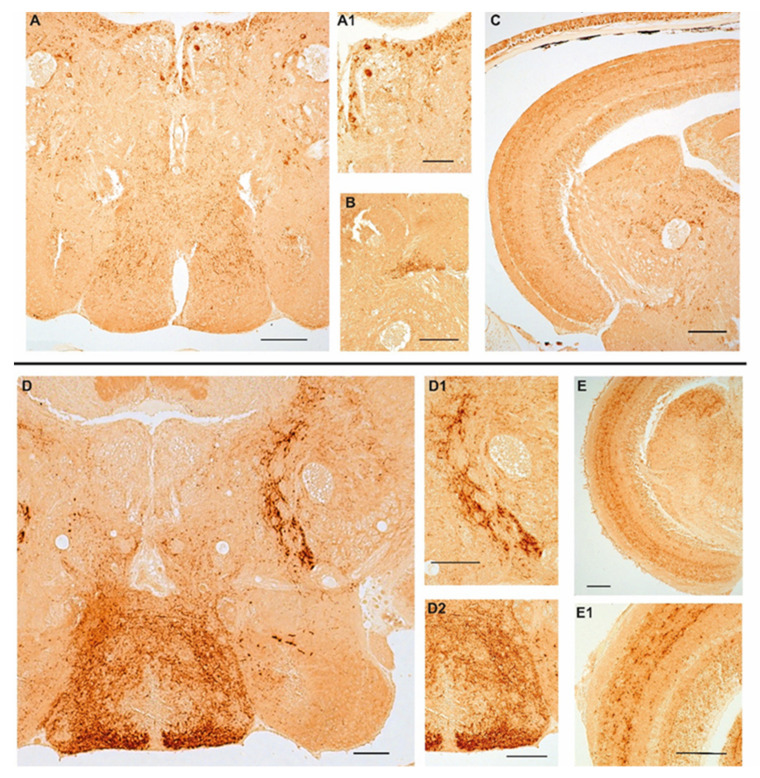
Age-related changes of NPY immunoreactivity in the brain of zebrafish. (**A**–**C**) Transverse sections of the brain of a 6-month-old zebrafish. (**A**) Overview of NPY immunoreactivity in the hypothalamic area (lateral and dorsal periventricular hypothalamus) and in the midbrain tegmentum of 6-month-old zebrafish. Positive neurons are distributed in the lateral hypothalamus, in the proximity of the preglomerular nucleus and along the ventricle. Abundant varicosities are distributed in the hypothalamus and over the entire tegmentum. (**A1**) Higher magnification of NPY positive neurons along the ventricle and in the proximity of the preglomerular nucleus. (**B**) Packed immunoreactive varicose fibers in the semicircular tori. (**C**) Overview of the optic tectum and the midbrain tegmentum displaying staining in a few neurons of the periventricular grey zone and in varicose fibers over the different layers of the optic tectum, numerous positive neurons along the ventricle and in the proximity of the preglomerular nucleus. (**D**–**E1**) Transverse sections of the brain of a 36-month-old zebrafish. (**D**) Overview of NPY immunoreactivity in the hypothalamic area (lateral and dorsal periventricular hypothalamus) and in the midbrain tegmentum of 36-month-old zebrafish. Few intensely immunostained neurons localized in the lateral and dorsal hypothalamus. Strong immunoreactivity in abundant varicosities distributed in the hypothalamus and projecting fibers toward the optic tectum and over the entire tegmentum. (**D1**) Higher magnification of projecting fibers toward the optic tectum. (**D2**) Higher magnification of abundant varicosities distributed in the hypothalamus. (**E**) Overview of NPY immunoreactivity in fibers in the optic tectum and in varicosities and neurons in the tegmentum. (**E1**) NPY immunostaining in the optic tectum layers (asterisks) and in a few cells of the periventricular grey zone. Scale bars 100 µm.

We previously documented the immunopositivity to NPY in the adult brain of turquoise killifish [30], mainly localized in the diencephalic region which is known to include the appetite and feeding control center. Similar to those observations, we confirmed that NPY was distributed in neurons of the ventral part of the telencephalon, cortical nucleus, along the margin of the ventral hypothalamus and in the dorsal part of hypothalamus as well as in the inferior lobe of hypothalamus, and in addition in the cells of the periventricular gray zone of turquoise killifish, either at 5 wph and 27 wph. It is noteworthy that in the same brain areas, the number of immunopositive neurons increased in the brain of old animals. Similarly, a wide distribution of typical NPY immunoreactive varicosities was detected along the neuroaxis that clearly increasing during aging (Figure S1). In the brain of 6- and 36-month-old zebrafish we observed immunoreactivity to NPY in few neurons localized in the ventral zones of telencephalon and in the preoptic area. We observed the majority of immunopositive neurons sparsely distributed in the hypothalamic area (lateral hypothalamus and dorsal periventricular hypothalamus) (Figure 2A,D,D2) and over the midbrain tegmentum (Figure 2A,D). In more detail, neurons were identified along the mesencephalic ventricle (Figure 1A), sparsely distributed in the layers of semicircular tori, around the preglomerular nucleus (Figure 2A,A1,C,D,D1). Typical immunopositive varicosities were abundantly distributed over the telencephalon, diencephalon, and tegmentum (Figure 2A–C,D–D2). Remarkably, immunoreactivity to NPY was detected in some neurons of the periventricular grey zone of the optic tectum (Figure 2C,E,E1) only at the border of the deep white and grey zone, while in the other layers of the optic tectum we observed immunoreactivity only in fibers (Figure 2C,E,E1). In the brain of 36-month-old animals, NPY immunolocalization was displayed in the same brain areas, although the immunostaining appeared stronger in more numerous neurons and neuronal projections than in the young specimens.

### 2.3. Immunoreactivity to NPY in the Gut of Turquoise Killifish and Zebrafish

**Figure 3:**
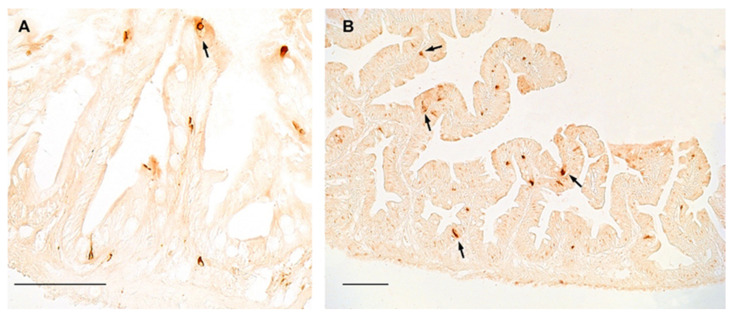
NPY immunoreactivity in the foregut of turquoise killifish. (**A**) Transverse section of the foregut of 5 wph turquoise killifish showing NPY immunopositivity in the cytoplasm of few enteroendocrine cells (arrow). (**B**) Transverse section of the foregut of 27 wph turquoise killifish showing NPY immunopositivity in the cytoplasm of enteroendocrine cells (arrows) over different villi.

In the gut of turquoise killifish, NPY was distributed in flask- or basket- shaped cells, featured by a broad base and an apical part that reaches the gut lumen, which are typical of the enteroendocrine cells, either in young (Figure 3A) and old (Figure 3B) animals, while slight immunostaining was reported in the myenteric plexus. However, our data did not display differences in the distribution and number of positive cells between young and old animals (Figure 3A,B).

**Figure 4:**
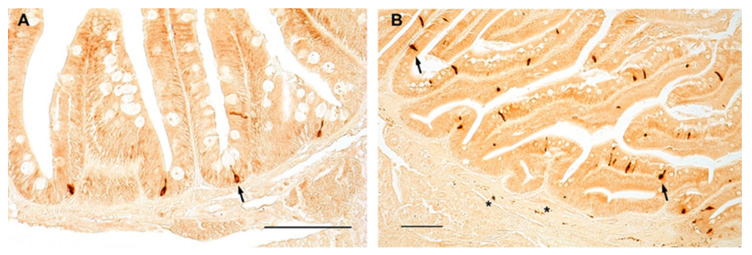
NPY immunoreactivity in the foregut of zebrafish. Transverse sections of the foregut of 6-month-old zebrafish showing NPY immunopositivity in the cytoplasm of enteroendocrine cells (arrows) (**A**,**B**) and in varicosities in the myenteric plexus (asterisks) (**B**).

Similarly, in zebrafish, NPY was distributed in the cytoplasm of clearly identifiable enteroendocrine cells of the foregut of young (Figure 4A) and old (Figure 4B) animals, with a slight increase in the foregut of old specimens. NPY immunostaining was clearly identified in the fibers of the myenteric plexus (Figure 4B), without age-related changes.

## 3. Discussion

In this study we investigated the age-related regulation of NPY, a neuropeptide involved in the regulation of food intake in fish [31] but also holding a pleiotropic role in several other vital functions, such as sleeping regulation [26], psychomotor activity [25,27], and spinal cord regeneration [28]. In a previous study we demonstrated that NPY mRNA increases in the brain of turquoise killifish during aging, thus hypothesizing a role of this neuropeptide, other than appetite control, in the brain aging of *N. furzeri* [30]. In this context, this study confirmed the age-increased levels of NPY in the brain of turquoise killifish and provided novel data concerning this regulation in another fish model, namely Danio rerio. Surprisingly, we observed that NPY increases in three-year-old zebrafish compared to young adult animals, suggesting a conserved regulation in the two fish species. In particular, in old zebrafish the increased levels of NPY mRNA in the brain correlated with the wider distribution of the NPY protein along the entire neuroaxis compared to young individuals. These findings are enhanced by the detection of a higher number of positive neurons and fibers in the brain of old specimens compared to those of young animals. Differently from *N. furzeri*, in which the wider expression of NPY in the old brain was due to a higher number of positive neurons localized also in non-diencephalic areas, such as the optic tectum and tegmentum [30], in the brain of old zebrafish we observed abundant positive neurons and fibers in the same areas, as described by Yokobori and colleagues [32]. More specifically, positive neurons were detected in the nuclei of the ventral zones of the telencephalon and the preoptic area as well as in the thalamic and hypothalamic areas along with the third ventricle, similar to previous descriptions [33]. Further, typical positive varicosities were identified along the entire neuroaxis. These results demonstrate that NPY expression increases in the brain of the two teleost fishes, which are both Actinopterigian despite belonging to two different families and although they display diverse key biological features [34] including lifespan and lifecycle [35]. Our findings correlate with previous studies suggesting the involvement of the NPY system in the aging process, particularly in the brain of mammals [36], and in association with neurodegenerative diseases through the activation of several mechanisms [7]. Cui and colleagues [28] reported that NPY was downregulated by aging in multiple brain regions, such as the cortex, hypothalamus, striatum, and spinal cord, while Michalkiewicz [8] showed improved resistance to stress exerted by the positive modulation of hemodynamic parameters and increased mean lifespan in transgenic rats overexpressing NPY [8] probably due to the modulation of the autophagy cascade in the CNS [37,38]. During caloric restriction, NPY levels increase in the hypothalamus, suggesting a role of NPY in autophagy modulation in hypothalamic neurons but also on whole-body aging (37). It has been shown that NPY receptor antagonists slowed the autophagic response to caloric restriction. Moreover, NPY enhanced autophagy in rodent hypothalamic neurons by stimulating Y1 or Y5 receptors through the combined activation of different pathways including class I phosphoinositide 3-kinase (PI3K), extracellular signal-regulated kinase (ERK), and protein kinase A (PKA) [37]. 

Further, it has recently been demonstrated that NPY delays the cellular aging phenotype associated to Hutchinson-Gilford Progeria Syndrome (HGPS), paving the way for the hypothesis that this neuropeptide may be exploited to block the premature onset of aging hallmarks in patients affected by HGPS by stimulating autophagy and decreasing progerin accumulation, rescuing nuclear abnormalities, and delaying cellular senescence [39]. In particular, the authors reported interesting findings displaying the NPY-induced enhancement of autophagic flux and progerin clearance in primary cultures of human dermal fibroblasts from patients affected by HGPS. In more detail, they showed that NPY preserves nuclear morphology and reduces the number of dysmorphic nuclei [39].

In this context, from which our study originated, it is worth nothing that several genes under positive selection, i.e., LMNA3 (LMNA(3of3), encoding a nuclear Lamin-A/C, whose mutations in humans lead to HPGS [40], have been annotated in the genome of turquoise killifish [41], highlighting the role of this model in brain aging research. Concerning our findings in the foregut, we observed a different age-related regulation in both species, where NPY mRNA levels significantly decreased during aging. Analyzing the expression pattern of the neuropeptide, we observed fewer immunopositive enteroendocrine cells lining the epithelium according to the results displayed in the duodenum of mice, in which Sandström and coworkers [42] reported that NPY decreased in animals at 12- and 24-months of age compared to those at 3-months old [42]. These data suggest a similar age-related gastro-intestinal regulation of NPY in vertebrates and could be relevant for translational studies. In addition, we observed that NPY is also localized in the fibers of the myenteric plexus of both species without any age-related changes, accordingly to recent data reported in mammals [43]. NPY immunoreactive neurons in the myenteric plexus did not change significantly in the duodenum of rats at diverse ages (1, 10, 20, 30, 60-days-old and 2-years-old), which is different from what happens in the submucosal plexus where they clearly decreased from young (30 days old) to old (2-years) rats [43]. It is noteworthy that we did not observe immunostaining in the submucous plexus either in turquoise killifish or in zebrafish. 

Our data document that NPY was expressed more in the CNS, despite the differences between young and old, than in the foregut, thus confirming the central regulation of this neuropeptide in the two fish species. More interestingly, functional studies are mandatory to dissect the significance of increased central levels of NPY during aging. This will open avenues for future studies on the pro and/or anti-aging role of this neuropeptide in the vertebrate brain. Even so, we have to recognize that the data we gained were not collected longitudinally since we based our study on evidence detected on different individuals analyzed at the two established time points. Therefore, we have no data about the expression levels of NPY in the two organs of interests in the same individual over time.

Moreover, considering that intestinal endocrine cells in zebrafish express orthologues for most known mammalian enteroendocrine hormones, including NPY [44], it may be interesting in future analyses to investigate whether the decrease in NPY associated with age is due to a reduced number of cells and/or to a reduced synthesis and storage of the neuropeptide in these cells.

In conclusion, in this study we reported for the first time the age-related regulation of NPY through the analysis of its expression in the brain and foregut in the shortest and in the longest-lived fish species. Both the chosen model organisms hold a well-recognized translational potential which allows the application of our findings to NPY modulation to enhance neuronal survival by impacting several aspects of age-related physiological and pathological processes.

## 4. Materials and Methods

### 4.1. Protocols

All experiments were performed:

- on group-housed *N. furzeri* belonging to the long-lived strain MZM 04/10 at the following time points: 5 weeks post hatching (wph), corresponding to a young adult at the age of the sexual maturity and 27 wph, corresponding to old individuals, at the onset of aging-related alterations [45]. Authorization for tissue sampling was carried out in accordance with the Italian legislative Decree (n°26/2014) (n° 6146A.N.4XE);
- on group-housed *D. rerio* belonging to the AB strain, at the following time points: 6 months post fertilization (mpf) (young-adult) and 36 mpf (old). Authorization for tissue sampling was carried out in accordance with the Italian legislative Decree (n°26/2014) (n° 6146A.N.WZ1).

### 4.2. Animal and Tissue Preparation

Fish at the selected time points were euthanized with an overdose of anesthetics, around 10 a.m. in a fasted state, to avoid the effects of circadian rhythms and feeding. Fish were placed for approximately 5–10 min in a methanesulfonate solution (MS-222, Tricane-S^®^, Western Chemical Inc., Gujarat, India) at a concentration of 1 mg/mL in buffered ethyl 3-aminobenzoate, until no vital signs (body and operculum movement, righting reflex) were observed. For RNA extraction, fresh brains and foreguts were dissected from the same animals, kept in sterile tubes with 500 µL of RNAlater (Qiagen), and stored at 4 °C for subsequent processing.

Morphological analyses were conducted on three specimens of either *N. furzeri* (frozen sections) or *D. rerio* (paraffin embedded sections). Tissues were rapidly excised and fixed in paraformaldehyde (PFA, 4% in diethylpyrocarbonate treated phosphate saline buffer (PBS)) overnight (ON) at 4 °C for *N. furzeri* while zebrafish tissues were fixed in Buin solution overnight. For cryostatic embedding, tissues were successively incubated in 30% and 20% sucrose solutions ON at 4 °C, embedded in cryomount and frozen at −80 °C. Then, serial transversal sections of 12 µm thickness for the brain and 10 µm thickness for the gut were cut with a Leica cryostat (Deerfield, IL, USA). For paraffin embedding, tissues were dehydrated in a graded ethanol series, embedded in paraffin, and serial transversal 7 µm thick sections were cut at the microtome.

### 4.3. RNA Extraction and the Reverse Transcription of cDNA Synthesis

Tissues were taken out of RNA later, cleaned with sterile papers and homogenized using a TissueLyzer II (Qiagen) at 20 Hz for 2–3 rounds × 1 min. Brains of 7 turquoise killifish and zebrafish (both young and old) and 7 guts of turquoise killifish and zebrafish (both young and old) were homogenized in 0.5 mL TriReagent (Sigma-Aldrich, Germany) followed by RNA isolation performed according to the manufacturer’s protocol. Total RNA was quantified using a NanoDrop ND-1000 Spectrophotometer (Thermo Scientific, Waltham, MA, USA) and RNA samples with a 260/280 nm ratio between 1.9 and 2.1 were selected for further analysis. Then 500 ng of each sample was retro-transcribed to cDNA in a total reaction volume of 20 µL, using the QuantiTect^®^ Reverse Transcription Kit (Qiagen, Hilden, Germany), and following the supplier’s protocol. Newly synthetized cDNAs were then diluted to a final volume of 200 µL with ultra-pure sterile water to an approximate final cDNA’s concentration of 40 ng/µL and stored at −20 °C.

### 4.4. Quantitative Real Time PCR

Primers were designed with Primer3 tool [46]. The primers used were for:

- *N. furzeri*:
  NPY fw CAGCCCTGAGACACTACATCA;
  NPY rev CTGCTCTCCTTCAGCAGCA;
- *D. rerio*:
  NPY fw ACAAAGCCCGACAACCCG;
  NPY rev AGCGCTTGACCTTTTCCCAT.

The correct amplicon size was verified by 1% agarose gel electrophoresis. Real-time PCR reactions were performed in 20 μL volume with 1 μL diluted cDNA using the Quantitect SYBR Green PCR kit (Qiagen) following the manufacturer’s instructions. A cDNA pool was serially diluted (from 80 to 2.5 ng per reaction) and used to create standard as well as melting curves and to calculate amplification efficiencies for the primer pair prior use for quantification. All reactions were performed in triplicate and negative (water) as well as genomic (without reverse transcriptase) controls were always included. Cycling parameters were as follows: 95 °C × 15 min, then 35 cycles at 94 °C × 15 s, 57 °C × 30 s, and 72 °C × 30 s (or 10 min initial denaturation at 95 °C followed by 40 cycles: 15 s in 95 °C, with 1 min amplification in 60 °C).

### 4.5. Statistical Analysis

Expression levels of NPY mRNA were analyzed by the ΔΔCt method and normalized to the housekeeping gene TATA-binding protein (TBP) for N. furzeri (TBP fw CGGTTGGAGGGTTTAGTCCT; TBP rev GCAAGACGATTCTGGGTTTG) and the housekeeping gene Ribosomal Protein S18 (RPS-18) for *D. rerio* (RPS-18 fw TGGTGTGGCTATGAACCCTG; RPS-18 rev TGGACGGTCTTTGTTCCTCG). Fold changes represent the difference in expression levels between young and old age NPY cDNAs, respectively, with young and old age housekeeping cDNAs. The relative ΔΔ curve threshold was built on fold changes values. One sample t-test and Wilcoxon *p*-value were calculated among young and old brains and young and old foreguts, as well as young brains versus young intestines and old brains versus old intestines. For this calculation, the Bonferroni correction was applied.

### 4.6. Immunohistochemistry

As previously describe [47,48] paraffin slides were deparaffinized in xylene and rehydrated in progressively diluted alcohols, whereas cryostatic slides were dried 2 h at room temperature (RT), placed in a bath of acetone 100% for 10 min at 4 °C, air-dried for a few minutes to optimize their fixation, and washed once in water and twice in 1× PBS. Then the slides were treated for 30 min with 3% H_2_O_2_ and, after washing with 1× PBS, were incubated in normal goat serum (abcam cat# ab138478, 1:5 in 1× PBS), at RT for 30 min. Incubation followed with a primary polyclonal antibody raised in rabbit against Neuropeptide Y (1:1000, abcam cat#ab30914), at 4 °C ON. Sections were then rinsed in 1× PBS for 15 min and then incubated with Ultrapolymer Goat anti-rabbit/mouse IgG (H&L) conjugated to HRP (ImmunoReagents cat# UNIHRP-015), for 1 h at RT. Immunoreactive sites were visualized using a fresh solution of 10 μg of 3,3′-diaminobenzidine tetrahydrochloride (DAB, Sigma-Aldrich, cat#D5905) in 15 mL of a 0.5 M Tris buffer.

### 4.7. Controls of Specificity

Positive controls were made by sections of a kids’ intestine (data not shown). Internal reaction controls were carried out by substituting primary antisera or secondary antisera with 1× PBS or normal serum in the specific step.

### 4.8. Image Acquisition

Images were observed and analyzed with Leica—DM6B (Leica, Wetzlar, Germany) and processed with LasX software. Digital raw images were optimized for image resolution, contrast, evenness of illumination, and background using Adobe Photoshop CC 2018 (Adobe Systems, San Jose, CA, USA). Anatomical structures were identified according to the adult *N. furzeri* brain atlas [49] and the Zebrafish virtual atlas [50].

## Data Availability

Not applicable.

## Supplementary Materials

The following supporting information can be downloaded at: https://www.mdpi.com/article/10.3390/ijms23073839/s1.
