# Supplementary material for: Central and Peripheral NPY Age-Related Regulation: A Comparative Analysis in Fish Translational Models
Source: Int J Mol Sci. 2022 Mar 30;23(7):3839. doi: 10.3390/ijms23073839 (PMC8998975; doi:10.3390/ijms23073839)
Supplement: Supplementary file 1 [file ijms-23-03839-s001.zip › ijms-1641329-supplementary.pdf]

Supplementary Materials

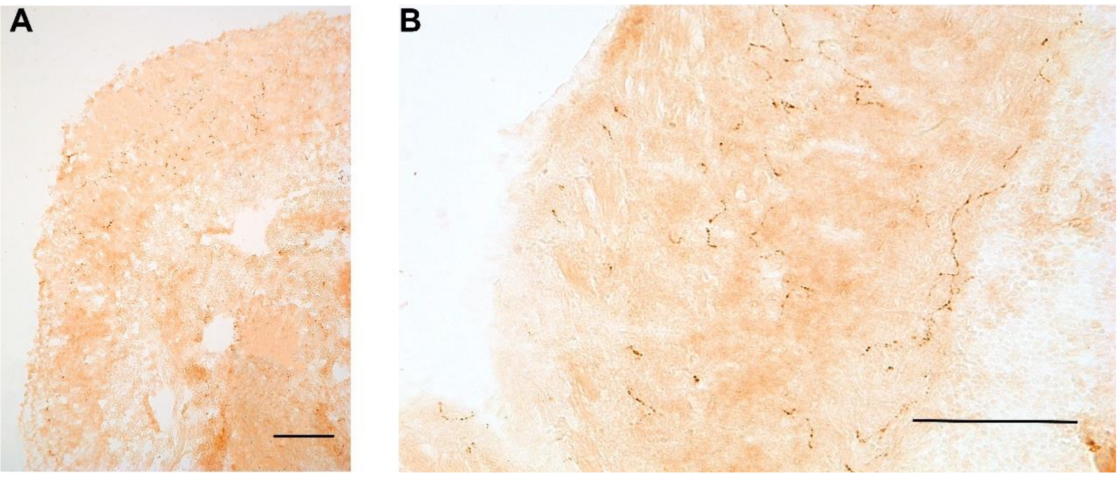

Figure S1: A wide distribution of typical NPY immunoreactive varicosities was detected along the neuroaxis clearly increasing over aging (**A**) Young brain *Nothobranchius furzeri* (**B**) Old brain *Nothobranchius furzeri*.
